# Impact of sarcopenia on functional and cognitive recovery in Caucasian post-stroke patients following rehabilitation

**DOI:** 10.3389/fnut.2025.1694609

**Published:** 2025-12-17

**Authors:** Alessandro Guerrini, Mariacristina Siotto, Alessio Fasano, Carola Cocco, Marco Germanotta, Valeria Cipollini, Laura Cortellini, Arianna Pavan, Stefania Lattanzi, Sabina Insalaco, Erika Antonacci, Elisabetta Ruco, Yeganeh Manon Khazrai, Irene Giovanna Aprile

**Affiliations:** 1 IRCCS Fondazione Don Carlo Gnocchi ONLUS, Florence, Italy; 2 Department of Science and Technology for Humans and the Environment, Università Campus Bio-Medico di Roma, Rome, Italy

**Keywords:** stroke, sarcopenia, functional recovery, cognitive recovery, body composition, EWGSOP2

## Abstract

**Background & aims:**

Sarcopenia, a progressive and generalized skeletal muscle disorder, significantly hinders post-stroke recovery. Existing research has focused exclusively on Asian populations, leaving effects in Caucasian cohorts largely unexplored. This study aims to evaluate the impact of sarcopenia, as defined by the European Working Group on Sarcopenia in Older People (EWGSOP2) criteria, on functional and cognitive recovery in subacute post-stroke patients undergoing a rehabilitation program.

**Methods:**

Eighty seven subacute post-stroke patients (71 [61–78] years; 42 women) were evaluated at admission (T0) and after 6 weeks of rehabilitation (T1). At T0, demographic, clinical, and nutritional data were collected, and sarcopenia was diagnosed. Functional and cognitive outcomes—including the modified Barthel Index (mBI), Fugl-Meyer Assessment for Upper Extremity (FMA-UE), Motricity Index for upper and lower limbs (MI-UE, MI-LE), Functional Ambulation Category (FAC), and Montreal Cognitive Assessment (MoCA)—were evaluated at both T0 and T1. Functional and cognitive recovery (ΔmBI, ΔFMA-UE, ΔMI-UE, ΔMI-LE, and ΔMoCA) were also assessed. Intra-group (T0 vs. T1) and inter-group comparisons (sarcopenic vs. nonsarcopenic patients) were then evaluated, and a Propensity Score Matching (PSM) analysis was used to adjust for baseline confounding factors.

**Results:**

Sarcopenic patients (*n* = 24; 14 women) showed poorer nutritional status and lower scores in all functional and cognitive measurements at T0 compared to their non-sarcopenic counterparts. Both groups improved significantly at T1 in mBI, FMA-UE, MI-UE, MI-LE, and FAC. However, even after PSM analysis, the sarcopenic patients exhibited lower FAC (0 [0–1] vs. 3 [1–3], *p* = 0.010) and lower mBI (40 [27–57] vs. 57 [47–72], *p* = 0.044) scores at T1, along with a reduced ΔmBI (6 [0–14] vs. 15 [8–21], *p* = 0.014).

**Conclusion:**

Our findings emphasize that sarcopenia negatively affects post-stroke recovery of independence and ambulation, highlighting the importance of early identification and targeted interventions in rehabilitation.

## 1 Introduction

Sarcopenia is a skeletal muscle disorder characterized by the progressive and generalized loss of muscle strength, mass, and function (1). First described by Irwin Rosenberg in 1989, it is now widely recognized as a major clinical concern due to its association with numerous adverse health outcomes, including impaired physical performance, mobility limitations, diminished quality of life alongside with an increased risk of falls, fractures, hospitalizations, and mortality (1, 2). It can be classified into primary sarcopenia, which occurs as a natural consequence of aging, and secondary sarcopenia, which results from reduced physical activity, inadequate nutrition, or as a consequence of various disease conditions, including stroke (3). In particular, post-stroke complications such as inflammation, hormonal dysregulations, muscle denervation, metabolic imbalances, and malnutrition can exacerbate a pre-existing sarcopenic state or, in some cases, even trigger its onset (4).

This stroke-related sarcopenia is particularly concerning in the context of post-stroke rehabilitation, as numerous studies demonstrated its negative impact across all phases of stroke recovery: post-stroke patients with sarcopenia or low muscle mass diagnosed in the acute phase exhibited poorer functional independence in Activity of Daily Living (ADL) at 3 months (5–8) and at 6 months (9) as measured by the modified Rankin Scale (mRS). During the subacute phase, sarcopenic patients have shown worse functional independence in ADL, as assessed by the Functional Independence Measure (FIM), compared to non-sarcopenic patients, at both admission (10, 11) and at the end of rehabilitation (11–14). Additionally, different studies reported that sarcopenia was also associated with reduced improvements in balance, ambulation, strength, and functional independence in ADL following the rehabilitation program (15, 16). A study involving 81 post-stroke patients during the chronic phase found that those with sarcopenia had a lower quality of life, as measured by the EQ-5D-3L questionnaire (17).

A major limitation of current research on stroke-related sarcopenia is that most available evidence derives from Asian cohorts, whereas data from European populations are largely lacking. This represents a critical gap for at least two reasons. First, body composition and muscle physiology differ substantially between Asian and Caucasian individuals (18, 19), with implications for both the diagnosis and the clinical consequences of stroke-related sarcopenia (1, 20, 21). Second, ethnic-specific dietary habits and lifestyle patterns, which differ markedly between Asian and Caucasian populations, may differentially influence the development of sarcopenia and its impact on post-stroke recovery (22–24). The European Working Group on Sarcopenia in Older People 2 (EWGSOP2) proposed diagnostic criteria and specific cut-off values derived from European reference populations (3). Similarly, the Asian Working Group for Sarcopenia (AWGS) recently established diagnostic thresholds specifically tailored to Asian populations, reflecting their typically smaller body frames, higher relative fat mass, and lower absolute muscle mass compared with Caucasians (19). To date, most studies on stroke-related sarcopenia in Asian populations have adopted AWGS-based criteria for muscle mass and strength (5, 6, 8, 10–15, 25, 26), while others applied different cut-off values (5, 7, 8, 27) resulting in heterogeneous definitions that limit cross-study comparability. The population-specific physiological and nutritional differences imply that findings from Asian stroke cohorts regarding the prognostic impact of sarcopenia on functional recovery cannot be directly applied to Caucasian post-stroke patients. Therefore, studies adopting the EWGSOP2 criteria are crucial to accurately evaluate this topic within European populations.

Within this context, we designed a study protocol involving a cohort of post-stroke patients in the subacute phase who were hospitalized for rehabilitation, with the aim of investigating the relationship between nutritional status, sarcopenia (diagnosed according to EWGSOP2 criteria), and recovery outcomes (NUTRISTROKE project). The analysis of the effect of sarcopenia on rehabilitation outcomes was conceived as a secondary objective within this protocol. In a preliminary study (28), we found that post-stroke patients with sarcopenia had poorer nutritional status, exhibited greater food waste during rehabilitation, and achieved smaller gains in ADL independence after a six-week rehabilitation program compared with non-sarcopenic patients.

In the present study, we aim to investigate, in the final cohort of the protocol, the impact of sarcopenia on independence in ADL, upper limb performance, muscle mass and strength, cognitive impairment, and functional ambulation at admission and discharge, as well as the variations in these parameters at the end of the rehabilitation program.

## 2 Materials and methods

### 2.1 Study design and participants

This prospective longitudinal cohort study (NUTRISTROKE, clinical trial identifier: NCT04923165) analyzed subacute post-stroke patients admitted to the “Santa Maria della Provvidenza” rehabilitation center of Fondazione Don Carlo Gnocchi in Rome between September 2020 and April 2023.

The inclusion criteria required patients to have experienced a first-ever ischemic or hemorrhagic stroke, confirmed through computed tomography (CT) or magnetic resonance imaging (MRI); be aged between 18 and 85 years; have a time since stroke onset of less than 6 months; and possess adequate cognitive and language abilities to follow instructions for clinical outcome assessments and provide informed consent.

Exclusion criteria included a history of prior stroke, cognitive or behavioral disorders, or reduced compliance that could interfere with active participation in rehabilitation or the ability to understand and sign informed consent. Additionally, patients with pacemakers were excluded due to potential interference with bioimpedance measurements.

Patients were evaluated at admission to the rehabilitation center (T0) and after completing a structured six-week rehabilitation program (T1). The study protocol was approved by the Ethics Committee of the Don Carlo Gnocchi Foundation in Milan, Italy, on February 12, 2020, with a non-substantial amendment on October 14, 2020 (Prot.n.22/2020/CE_ FdG/FC/SA_14/10/20). All participants provided written informed consent after receiving a comprehensive explanation of the study’s objectives and rehabilitation procedures.

### 2.2 Rehabilitation program

Patients underwent a structured six-week rehabilitation program, consisting of conventional physical therapy sessions lasting 45 min per day, 6 days a week. The rehabilitation treatment targeted both upper and lower limb recovery and included a combination of passive, active-assisted, and active mobilization exercises. Specific techniques focused on reducing hypertonia, facilitating muscle fiber recruitment, and enhancing motor control and sensorimotor function. For lower limb exercises, the rehabilitation included balance exercises, gait training, postural passages and transfers, proprioceptive training and motion pattern walking training.

For lower limb exercises, the rehabilitation included task-oriented exercises such as grasping, reaching, and pinching movements, as well as occupational therapy (e.g., dressing, bathing, brushing/combing hair, depending on subject ability). Additionally, all patients underwent robotic-assisted therapy for the upper limb, conducted five times per week, with each session lasting 45 min. This therapy involved four advanced robotic devices: (i) Amadeo (Tyromotion, Austria) a robotic system enabling sensorimotor hand exercises in passive, active, and active-assistive modes.; (ii) Pablo (Tyromotion, Austria) a sensor-based system supporting unimanual and bimanual movements of the shoulder, elbow, and wrist, as well as trunk control exercises; (iii) Motore (Humanware, Italy), a robotic device facilitating passive, active, and active-assistive planar shoulder and elbow joints movements; (iv) Diego (Tyromotion, Austria), which is an electromechanical system designed for three-dimensional, unimanual, and bimanual shoulder movements with arm weight support. During robotic therapy, patients performed motor and cognitive tasks while receiving real-time visual and auditory feedback to optimize movement execution and promote neuroplasticity (29).

### 2.3 Demographic, clinical and nutritional assessment

Demographic and clinical data were collected at T0. To assess the cumulative medical burden of patients, the 56-point Cumulative Illness Rating Scale (CIRS) was administered (30). At T0, the presence of dysphagia, comorbidities, and time since stroke onset were recorded.

Anthropometric parameters were assessed by investigators trained in standardized measurement methods. Body mass was measured at T0 and T1: patients who were able to stand were weighed using a calibrated scale (Seca 750, Seca Hamburg, Germany), while those unable to stand were assessed using a chair weighing scale (Wunder DE5, Wunder Sa.Bi. S.r.l; Milan, Italy). Both weight assessment tools had a sensitivity of 0.1 kg. Height was measured at T0 for ambulatory patients using a stadiometer, with data recorded in meters (m) to the nearest 0.1 cm. For non-ambulatory patients, height was estimated using the knee height method (31). The Body Mass Index (BMI, kg/m^2^) at T0 and T1 was subsequently derived.

Hematochemical analyses were conducted at T0 using an integrated analytical photometer (Free *Carpe Diem*, Diacron International S.r.l., Grosseto, Italy). Blood sample collection, processing, and storage followed standardized protocols. The determination of serum concentration of glucose (mg/dL), of total cholesterol (mg/dL), of HDL cholesterol (mg/dL), of triglycerides (mg/dL), and of albumin (g/L), was performed according to established procedures, as detailed in our previous study (32). Serum Creatinine concentration (mg/dL) was determined by Jaffe’s reaction where creatinine produces quantitatively an orange color with picric acid in alkaline medium (33). At T0, patients’ nutritional status was assessed using the Mini Nutritional Assessment Short-Form (MNA-SF®), the Geriatric Nutritional Risk Index (GNRI), and Phase Angle (PhA) measured with Bioelectrical Impedance Analysis (BIA). MNA-SF® is a brief, validated tool for malnutrition screening in geriatric populations (34). It consists of six items evaluating food intake decline, weight loss, mobility, psychological stress or acute disease, neuropsychological problems, and BMI, with each item scored from 0 to 3. The total score ranges from 0 to 14, with lower values indicating a higher risk of malnutrition. The GNRI is a biomarker assessing the risk of nutritional complications (35), calculated from serum albumin levels and ideal body weight, as explained previously (28). The BIA-derived PhA was assessed using a phase sensitive bioimpedance devices with a foot-to-hand configuration, operating at a fixed frequency of 50 kHz (BIA 101 Anniversary Sport Edition, Akern, Firenze, Italy). The full BIA assessment procedure is detailed in our previous study (36).

Patient food intake during the rehabilitation period was assessed through the visual estimation “plate waste” method, as previously detailed (32). Trained nurses and speech therapists recorded the amount of food wasted at each meal (breakfast, lunch, and dinner) 6 days per week over the six-week rehabilitation period, corresponding to a total of 108 meals per patient. A score from 0 to 4 was assigned on a 5-point scale (0 = not wasted; 1 = ¼, 2 = ½, 3 = ¾, or all wasted) (37, 38). For each patient, the average percentage of food wasted was then calculated for all 108 meals consumed. During the six-week rehabilitation period, patients followed a diet consisting of meals prepared by the dietary service of our rehabilitation center, in accordance with the Italian Nutritional Guidelines (39).

### 2.4 Sarcopenia assessment

Sarcopenia was evaluated according to the EWGSOP2 criteria (3), by the simultaneous presence of low values of muscle mass strength and low muscle mass quantity. Severe sarcopenia was determined through physical performance evaluation. Muscle mass strength was measured using a hand-held digital dynamometer (Citec, C.I.T Techincs, Haren, Netherlands). Specifically, the maximum isometric strength of the non-hemiparetic hand and forearm muscles was recorded (40). Patients were assessed while seated, with their elbows flexed at 90°, shoulders adducted, and forearms in a neutral position without external support. The maximum hand grip strength (HG), expressed in kilograms (kg), was taken from three maximal isometric contractions performed with brief rest intervals. According to EWGSOP2 guidelines, probable sarcopenia was defined as handgrip strength below 27 kg in men and below 16 kg in women. Muscle mass quantity was estimated by calculating the Appendicular Skeletal Muscle Mass (ASMM) divided by the patient’s height squared (ASMM/h^2^; kg/m^2^), using a specific population and BIA validated predictive equation (41). According to EWGSOP2 thresholds, low muscle mass quantity was defined as <5.5 kg/m^2^ for women and <7 kg/m^2^ for men. Severe sarcopenia was diagnosed in individuals with low physical performance, determined by a gait speed below 0.8 m/s. Gait speed was assessed using the 10-meter walk test (42). Patients who were unable to stand were classified as having poor physical performance. Clinicians and therapists who conducted clinical, functional, and cognitive assessment at both T0 and T1 were not aware of the participants’ sarcopenia status, as sarcopenia analysis was performed after data collection.

### 2.5 Functional and cognitive outcome measures

In our study, patients were evaluated both at T0 and at T1 using different clinical outcome measures to assess their Independence in Activity of Daily Living (ADL), limb performance, limb strength, functional ambulation, and cognitive abilities.

The independence in ADL was assessed by the modified Barthel Index (mBI). The mBI consists of 10 items assessing a patient’s ability to perform essential daily activities, including feeding, personal hygiene, dressing, bathing, bladder and bowel control, toilet transfers, stair climbing, and ambulation or wheelchair use. Each item is assigned a numeric score based on the level of assistance required, with a total possible score of 100 points. Lower scores indicate greater mobility and self-care impairments, whereas higher scores reflect greater functional independence (43).

Upper limb performance was evaluated with the Fugl-Meyer Assessment for the Upper Extremity scale (FMA-UE) that is designed to evaluate motor and sensory function of the hemiparetic arm (44). The FMA-UE consists of 33 items, each scored on a three-point scale: 0 (cannot perform), 1 (performs partially), and 2 (performs fully). The total score ranges from 0 to 66.

To evaluate the strength of the limbs affected by hemiparesis following stroke, the Motricity Index of the Upper (MI-UE) and Lower (MI-LE) extremity were employed (45, 46). For the MI-UE, assessments included pinch grip, elbow flexion, and shoulder abduction of the affected arm. For the MI-LE, ankle dorsiflexion, knee extension, and hip flexion of the affected leg were evaluated. The total score for both MI-UE and MI-LE ranges from 1 to 100.

Functional ambulation was assessed using the Functional Ambulation Category (FAC), which classifies walking ability into six levels based on the degree of assistance required, ranging from 0 (non-functional ambulation) to 5 (fully independent ambulation) (47).

Cognitive abilities were examined using the Italian version of the Montreal Cognitive Assessment (MoCA). The MoCA includes various subtests designed to explore abstraction, set-shifting, and cognitive flexibility. The total MoCA score ranges from 0 to 30, with higher scores indicating better overall cognitive performance. The final MoCA value was calculated using adjustment grids according to age and education for MoCA total and subtest raw scores (48).

All assessments were conducted by experienced clinicians specifically trained in the administration and scoring of the instruments used in this study. Specifically, Physiotherapists specialized in neurorehabilitation performed the motor and functional evaluations (mBI, FMA-UE, MI-LE, MI-UE, FAC) following standardized procedures, while MoCA was administered by trained neuropsychologists and speech therapists.

### 2.6 Statistical analysis

The sample size was established *a priori* for the primary outcome of NUTRISTROKE protocol, which aimed to investigate the possible correlation between nutritional status indices and rehabilitation outcome. Specifically, the G*Power software was employed (v. 3.1.9.7), assuming an expected correlation of *r* = 0.3 between baseline nutritional indicators and functional recovery outcomes, a two-tailed *α* = 0.05, and a statistical power (1–*β*) = 0.80, the required sample size was estimated to be 85 participants.

The demographic and clinical characteristics of the patients were presented using numerical data expressed as mean and standard deviation for normally distributed data, and as median and interquartile range (25th, 75th percentiles) for non-normally distributed data. Categorical data were expressed as counts and percentages.

The Shapiro–Wilk test was used to assess the normality of data distributions. As the assumption of normality was violated, non-parametric tests were subsequently applied.

At T0, we compared demographic, clinical, and nutritional data, as well as functional and cognitive status data between women and man using the Mann–Whitney U test for ordinal or continuous data, and with chi-squared test for categorical variables. The same analyses were conducted to compare data between sarcopenic and non-sarcopenic individuals.

The Wilcoxon signed-rank test was performed separately for sarcopenic and non-sarcopenic individuals in order to evaluate intra-group changes over time (T0 and T1) in functional and cognitive outcomes, as well as in muscle mass strength (HG) and quantity (ASMM/h2).

To investigate the effect of sarcopenia on functional and cognitive outcomes, we compared these outcomes between sarcopenic and non-sarcopenic groups at the end of rehabilitation (T1) using the Mann–Whitney U test. To account for potential confounding variables, the same analysis was repeated after creating two groups of sarcopenic and non-sarcopenic patients matched considering baseline differences, through a Propensity Score Matching (PSM) analysis (49). Specifically, we considered as potential confounders variables that may indirectly affect recovery, including age, BMI, baseline functional status (mBI, FMA-UE, MI-UE, MI-LE, and MoCA at T0), sex, days from stroke onset, and comorbidity (assessed by the CIRS comorbidity index). To maintain the robustness and reliability of the PSM model and to avoid overfitting, only those variables that were significantly different between the two groups at baseline were included in the PSM. For baseline functional and cognitive variables, we computed a composite Z-score, obtained as the mean of the Z-scores of each measure. The composite Z-score, along with the other selected variables, was then fitted in the matching procedure. The MatchIt package in RStudio (RStudio 2024.4.2.764, PBC, Boston, MA) was employed for this analysis.

To evaluate the recovery in functional and cognitive outcomes, we calculated the changes from baseline of the following factors: independence in ADL (∆mBI = mBIT1 − mBIT0), upper limb performance (∆FMA-UE = FMA-UE T1 − FMA-UE T0), upper (∆MI-UE = MI-UE T1 − MI-UE T0) and lower (∆MI-LE = MI-LE T1 − MI-LE T0) limb strength, and cognitive abilities (∆MoCA = MoCA T1 − MoCA T0). Delta scores were also computed for HG and ASMM/h^2^. We then compared these recovery outcomes between sarcopenic and non-sarcopenic individuals in both unmatched and matched groups using the Mann–Whitney U test.

For all the statistical analyses, a *p* value below 0.05 was considered significant. Statistical analysis was performed using SPSS (IBM SPSS Statistics for Windows, Version 28.0. Armonk, NY: IBM Corp).

## 3 Results

### 3.1 Participants and baseline characteristics

In this longitudinal study, 91 subacute stroke patients who met the inclusion criteria and provided written informed consent were included and analyzed at baseline. Of those, four patients did not complete the final assessment due to reasons unrelated to the study protocol and were therefore considered dropouts. Consequently, the final sample consisted of 87 patients (women *n* = 42). Baseline characteristics for the whole group and divided by gender, including anthropometric measurements, demographic and clinical features, nutritional status assessment, sarcopenia assessment and disability assessment, are shown in Table 1. We identified 39 probable cases of sarcopenia (women *n* = 18), of which 24 were confirmed (women *n* = 18). Severe sarcopenia was found in 23 sarcopenic patients (96%). There were no significant differences in the prevalence of sarcopenia between the sexes. Three patients were excluded from the FAC analysis due to missing data.

**Table 1:** Baseline (T0) characteristics of the group (*n* = 87) and of women (*n* = 42) and men (*n* = 45).

Baseline characteristics	Whole group (*n* = 87)	Women (*n* = 42)	Men (*n* = 45)	*p-value*
Age (years)	71 [61–78]	74 [66–78]	68 [56–76]	0.110
Anthropometric measurements				
Weight (kg)	68.0 [58.1–78.2]	60.7 [54.0–1.65]	73.0 [63.0–85.5]	<0.001***
Height (m)	1.61 [1.55–1.65]	1.61 [1.55–1.65]	1.70 [1.66–1.68]	<0.001***
BMI (kg/m^2^)	24.2 [22.0–27.3]	23.2 [21.0–26.7]	25.2 [23.2–28.0]	0.058
Index stroke type				
Ischemic	66 (76%)	33 (78%)	33 (73%)	0.412
Hemorrhagic	21 (24%)	9 (22%)	12 (27%)	
Smoking habit (smokers and ex-smokers)	45 (52%)	27 (64%)	18 (40%)	0.165
Comorbidities				
Hypertension	74 (85%)	35 (83%)	39 (87%)	0.568
Type 2 diabetes	26 (30%)	11 (26%)	15 (33%)	0.663
Dyslipidemia	37 (43%)	18 (43%)	19 (42%)	0.467
Heart disease	12 (14%)	3 (7%)	11 (24%)	0.028*
Dysphagia	29 (33%)	16 (38%)	13 (29%)	0.363
Cumulative Illness Rating Scale (CIRS)				
CIRS severity	2.2 [2.0–2.5]	2.3 [2.0–2.5]	2.2 [2.0–2.4]	0.309
CIRS comorbidity	5.0 [4.0–7.0]	5.5 [4.3–7.0]	5.0 [4.0–6.0]	0.570
Days from stroke onset to enrollment	87 [72–118]	91 [74–127]	82 [71–102]	0.224
Hematochemical analyses				
Glucose (mg/dL)	99 [81–121]	99 [82–128]	95 [81–115]	0.312
Cholesterol (mg/dL)	120 [95–146]	117 [98–158]	122 [90–141]	0.199
HDL Cholesterol (mg/dL)	52 [44–63]	58 [48–62]	49 [36–59]	0.006**
Triglycerides (mg/dL)	112 [83–147]	107 [81–144]	119 [83–148]	0.415
Albumin (g/L)	3.8 ± 0.5	3.7 ± 0.5	3.9 ± 0.5	0.351
Creatinine (mg/dL)	1.0 [0.9–1.3]	0.9 [0.8–1.1]	1.2 [1.0–1.5]	<0.001***
Nutritional status assessment				
GNRI	105 ± 13	103 ± 14	106 ± 11	0.292
MNA-SF®	8 ± 2	7 ± 2	8 ± 2	0.592
PhA (degree)	4.7 [3.9–5.6]	4.3 [3.6–5.2]	5.1 [4.2–5.8]	0.006**
Sarcopenia assessment				
Hand grip strenght test (kg)	22 [15–32]	16.3 [12.2–22.0]	27.6 [21.0–36.8]	<0.001***
ASMMI (kg/m^2^)	6.7 [5.8–7.8]	5.8 [5.3–6.7]	7.5 [6.7–8.5]	<0.001***
Probable sarcopenia (*n*, %)	39 (45%)	18 (43%)	21 (48%)	0.650
Sarcopenia confirmed (*n*, %)	24 (28%)	14 (33%)	10 (22%)	0.247
Severe sarcopenia (*n*, %)	23 (96%)	13 (93%)	10 (100%)	0.388
Independence in Activity of daily living (ADL)				
Modified Barthel Index T0 (0–100)	40 [32–54]	40 [29–50]	41 [34–62]	0.144
Upper limb performance				
Fugl-Meyer T0 (0–66)	29 [6–51]	15 [4–48]	31 [11–55]	0.067
Upper and lower limbs strength				
Motricity Index Upper Extremities (1–100)	50 [19–77]	40 [1–67]	58 [26–77]	0.143
Motricity Index Lower Extremities (1–100)	49 [33–76]	43 [24–76]	58 [34–76]	0.246
Functional Ambulation Category (0–5)	1 [0–2]	0 [0–2]	1 [1–3]	0.132
Montreal Cognitive Assessment (0–30)	19 [15–24]	17 [14–24]	21 [17–25]	0.024

Data are reported as mean ± standard deviation for normally distributed variables, median and interquartile range (25th–75th percentiles) for non-normally distributed variables, or number and percentage (%) for categorical variables. *p*-values refer to the Mann–Whitney U test or the chi-squared test, as appropriate. *p-value < 0.05; **p-value < 0.01; ***p-value < 0.001.

### 3.2 Differences in baseline characteristics and food consumption between sarcopenic and non-sarcopenic patients

Table 2 shows the baseline characteristics of patients, classified according to the presence or absence of sarcopenia.

**Table 2:** Baseline characteristics in sarcopenic (*n* = 24) and non-sarcopenic (*n* = 63) participants.

Baseline characteristics	Sarcopenic *n* = 24	Non-sarcopenic *n* = 63	*p*-value
Age	75 [69–79]	68 [57–76]	0.015*
Gender			
Women	14 (58%)	28 (44%)	0.247
Men	10 (42%)	35 (56%)	
Anthropometric measurements			
Weight (kg)	56.7 [51.1–64.2]	72.3 [62.4–82.7]	<0.001***
Height (m)	1.62 [1.59–1.69]	1.68 [1.60–1.75]	0.083
BMI (kg/m^2^)	22.0 [19.5–24.1]	25.3 [22.9–28.2]	<0.001***
Index stroke type			
Ischemic	21 (88%)	45 (71%)	0.117
Hemorrhagic	3 (12%)	18 (29%)	
Smoking habit (smokers and ex-smokers)	11 (46%)	34 (54%)	0.469
Comorbidities			
Hypertension	17 (71%)	57 (90%)	0.022*
Type 2 Diabetes	4 (17%)	22 (35%)	0.096
Dyslipidemia	8 (33%)	29 (46%)	0.284
Heart disease	2 (8%)	12 (19%)	0.224
Dysphagia	14 (58%)	15 (29%)	0.002**
Cumulative Illness Rating Scale (CIRS)			
CIRS severity	2.3 [2.1–2.5]	2.2 [2.0–2.5]	0.181
CIRS comorbidity	6.0 [5.0–7.0]	5.0 [4.0–6.0]	0.137
Days from stroke onset to enrollment	89 [77–100]	82 [69–124]	0.750
Hematochemical analyses			
Glucose (mg/dL)	96 [82–124]	99 [81–121]	0.729
Cholesterol (mg/dL)	100 [85–139]	128 [103–149]	0.025*
HDL Cholesterol (mg/dL)	49 [40–63]	53 [44–63]	0.540
Triglycerides (mg/dL)	94 [76–120]	120 [94–151]	0.030*
Albumin (g/L)	3.6 ± 0.6	3.9 ± 0.4	0.021*
Creatinine (mg/dL)	0.9 [0.8–1.1]	1.1 [0.9–1.3]	0.032*
Nutritional status			
GNRI	96.3 ± 11.8	107.7 ± 11.5	<0.001***
MNA-SF®	6.2 ± 2.7	8.0 ± 2.0	<0.001***
PhA (degree)	4.1 [3.5–4.7]	5.0 [4.2–5.8]	0.002**

Data are reported as mean ± standard deviation for normally distributed variables, median and interquartile range (25th–75th percentiles) for non-normally distributed variables, or number and percentage (%) for categorical variables. *p*-values refer to the Mann–Whitney U test or the chi-squared test, as appropriate. *p-value < 0.05; **p-value < 0.01; ***p-value < 0.001.

**Figure 1:**
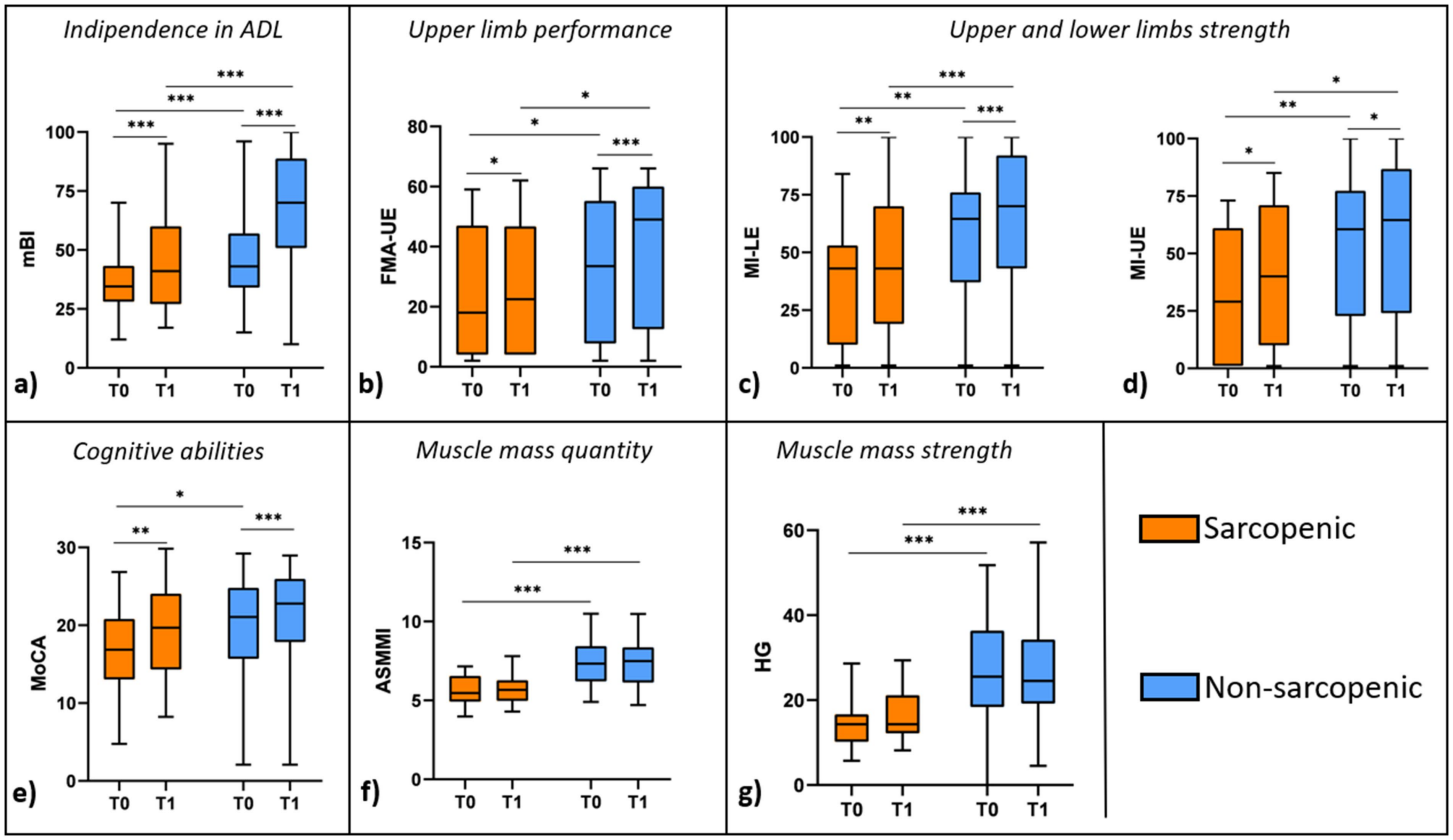
Box-plot diagram showing the intergroup (sarcopenics vs. non-sarcopenics) and intragroup (T0 vs. T1) comparisons for: **(a)** Independence in ADL (Modified Barthel Index, mBI); **(b)** Upper limb performance (Fugl-Meyer Assessment for the Upper Extremity, FMA-UE); **(c)** Lower limb strength (Motricity Index for the Lower Extremity, MI-LE); **(d)** Upper limb strength (Motricity Index for the Upper Extremity, MI-UE); **(e)** Cognitive abilities (Montreal Cognitive Assessment, MoCA); **(f)** Muscle mass quantity (Appendicular Skeletal Muscle Mass Index, ASMMI); **(g)** Muscle mass strength (Hand Grip Strength, HG). Median values, upper and lower quartiles are displayed for each variable. *, **, *** refer to the statistically significant differences (*p < 0.05, **p < 0.01; ***p < 0.001), according to Wilcoxon signed rank test or the Mann–Whitney test, as appropriated.

At T0, sarcopenic patients were older (75 [69–79] vs. 68 [57–76] years, *p* = 0.015) and had lower BMI values (22.0 [19.5–24.1] vs. 25.3 [22.9–28.2], *p* < 0.001). Additionally, the sarcopenic group exhibited lower GNRI values (96.3 ± 11.8 vs. 107.7 ± 11.5, *p* < 0.001), lower PhA values (4.1 [3.5–4.7] vs. 5.0 [4.2–5.8], *p* = 0.002), and lower MNA-SF® scores (6.2 ± 2.7 vs. 8.0 ± 2.0, *p* < 0.001) compared with the non-sarcopenic group. Dysphagia was significantly more prevalent in sarcopenic patients (58% vs. 29%, *p* = 0.002), while hypertension was more prevalent in non-sarcopenic subjects (90% vs. 71%, *p* = 0.022). Comparing hematochemical parameters in the two groups, sarcopenic patients had lower levels of cholesterol (100 [85–139] mg/dL vs. 128 [103–149] mg/dL, *p* = 0.025), triglycerides (94 [76–120] mg/dL vs. 120 [94–151] mg/dL, *p* = 0.030) albumin (3.6 ± 0.6 g/L vs. 3.9 ± 0.4 g/L, *p* = 0.021), and creatinine (0.9 [0.8–1.1] mg/dL vs. 1.1 [0.9–1.3] mg/dL, *p* = 0.032). At admission, all functional and cognitive outcomes were lower in sarcopenic patients compared to non-sarcopenic ones (Figure 1). Regarding functional ambulation, the prevalence of patients unable to ambulate at admission (FAC = 0) was higher in sarcopenic patients with respect to non-sarcopenic ones (78% vs. 34%, *p* < 0.001).

When comparing food consumption between sarcopenic and non-sarcopenic patients, sarcopenic individuals showed a higher percentage of food waste during the rehabilitation period (28 [17–40] % vs. 13 [7–24] %, *p* = 0.005).

### 3.3 Impact of sarcopenia on functional and cognitive outcomes: changes following rehabilitation treatment

Several functional and cognitive outcomes improved after rehabilitation, in both sarcopenic and non-sarcopenic groups (Figure 1). Specifically, mean mBI scores significantly increased from T0 to T1 in both sarcopenic patients (41 [27–58] vs. 35 [28–42], *p* = 0.004) and non-sarcopenic patients (70 [52–88] vs. 43 [35–57], *p* < 0.001). Similar improvements were observed in FMA-UE (sarcopenic: 23 [5–46] vs. 18 [4–42], *p* = 0.015; non-sarcopenic: 49 [13–59] vs. 34 [9–55], *p* < 0.001), MI-LE (sarcopenic: 43 [22–70] vs. 43 [15–53], *p* = 0.008; non-sarcopenic: 70 [43–91] vs. 65 [38–76], *p* < 0.001), MI-UE (sarcopenic: 40 [10–66] vs. 29 [6–56], *p* = 0.030; non-sarcopenic: 65 [24–86] vs. 61 [24–77], *p* = 0.011) and MoCA (sarcopenic: 20 [15–24] vs. 17 [13–21], *p* = 0.002; non-sarcopenic: 23 [18–26] vs. 21 [16–25], *p* < 0.001). On the contrary, ASMMI and HG did not improve after rehabilitation in either group.

**Figure 2:**
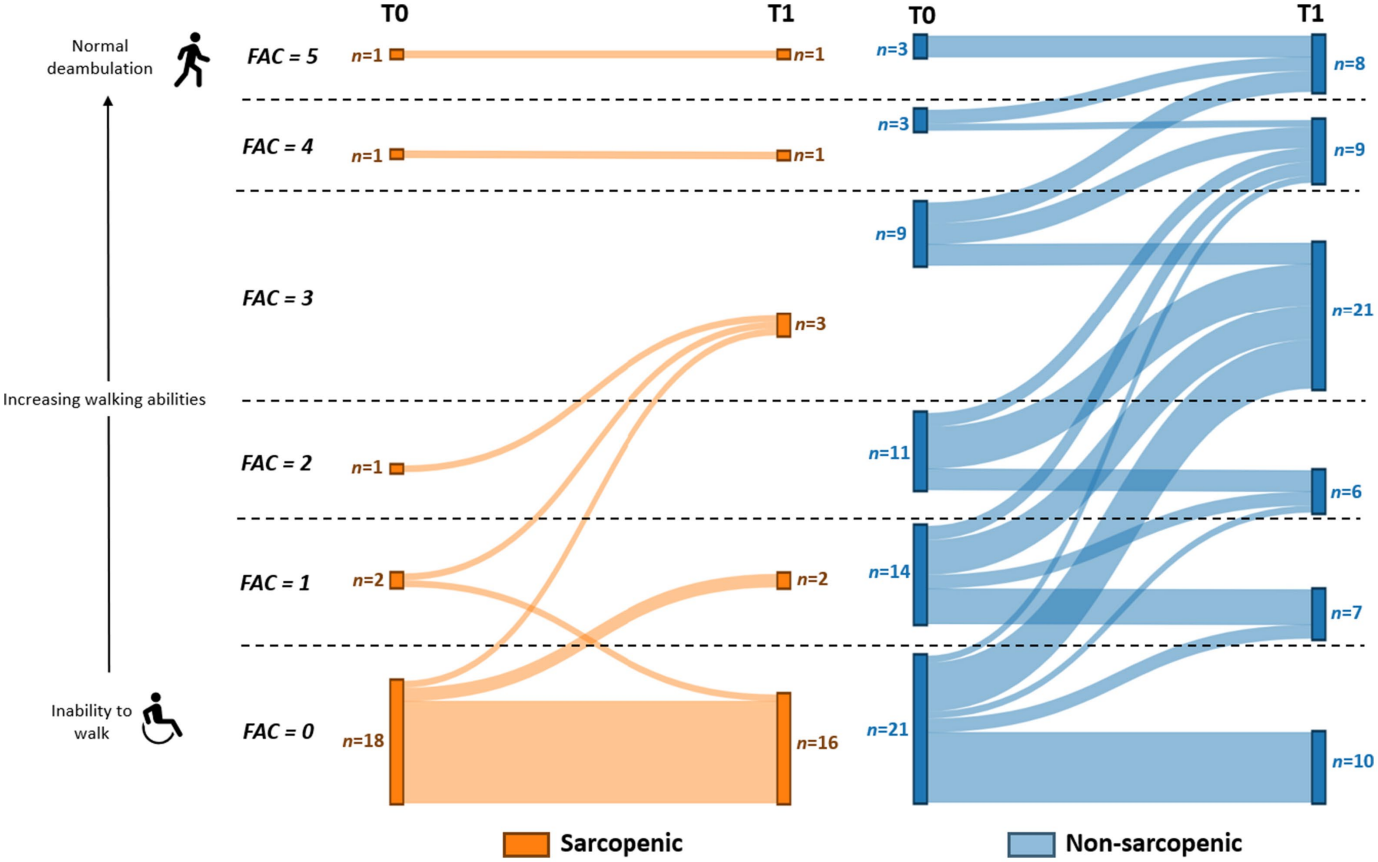
Sankey diagram illustrating the recovery of sarcopenic and non-sarcopenic patients in functional ambulation during rehabilitation period. The diagram visualizes the flow and transitions of patients across different Functional Ambulation Categories (FAC) from T0 to T1 (after 6-weeks of rehabilitation).

Moreover, non-sarcopenic participants exhibited a significant increase in FAC scores at T1 compared to T0 (3 [1–4] vs. 1 [0–3], *p* < 0.001), whereas no significant improvement was observed in the sarcopenic group (0 [0–1] vs. 0 [0–0], *p* = 0.106). While 59% of non-sarcopenic patients reached higher FAC levels after rehabilitation, only 5 sarcopenic patients (24%) improved their ambulation, while 1 sarcopenic patient experienced a decline (Figure 2). Notably, among patients unable to walk at admission (FAC = 0, sarcopenic: *n* = 18, non-sarcopenic: *n* = 21), 52% of the non-sarcopenic patients reached higher FAC levels after rehabilitation, whereas only 17% of the sarcopenic group demonstrated a similar improvement. It is important to emphasize that we did not find significant differences in age between the sarcopenic and non-sarcopenic unable to walk at admission (FAC = 0).

All outcome measures at T1 differed significantly between the two groups, except for MoCA (Figure 1).

Regarding PSM analysis, age, BMI, and baseline functional and cognitive scores were included as covariates, as these variables differed between sarcopenic and non-sarcopenic patients at T0 (Table 2). In contrast, sex, days from stroke onset, and CIRS comorbidity at T0 were not included. After PSM, we obtained a balanced sample of 46 patients, consisting of 23 sarcopenic and 23 non-sarcopenic individuals, with an equal proportion of women (50%) in each group (Supplementary Figure S1). No differences were found between the matched groups in terms of age, BMI, functional and cognitive scores at baseline, or presence of comorbidities (Supplementary Table S1). When we compared the two matched groups in terms of functional and cognitive outcomes at T1, only the mBI and FAC were different between sarcopenic and non-sarcopenic patients (Supplementary Table S2).

### 3.4 Differences in recovery outcomes between sarcopenic and non-sarcopenic patients

Table 3 presents the differences in recovery outcomes between sarcopenic and non-sarcopenic individuals in the total unmatched sample. The recovery of independence in ADL, as indicated by the ΔmBI score, was higher in the non-sarcopenic compared to sarcopenic patients (17 [6–29] vs. 7 [0–16], *p* = 0.004). Conversely, no significant differences were found between the two groups in the recovery of upper limb performance, upper and lower limbs strength, or cognitive abilities. As expected, ∆ASMMI and ∆HG did not differ between the groups. Notably, after matching for baseline characteristics, ΔmBI still emerged as the only recovery outcomes that remained lower in sarcopenic patients (6 [0–14] vs. 15 [8–21], *p* = 0.014).

**Table 3:** Recovery outcome differences between sarcopenic (*n* = 24) and non-sarcopenic (*n* = 63) participants.

Recovery outcome	Sarcopenic *n* = 24	Non-sarcopenic *n* = 63	*p-value*
∆mBI	7 [0–16]	17 [6–29]	0.004**
∆FMA-UE	2 [0–6]	4 [1–8]	0.313
∆MI-LE	5 [0–14]	0 [0–14]	0.840
∆MI-UE	5 [0–18]	8 [0–16]	0.472
∆MoCA	2 [1–4]	1 [0–3]	0.218
∆ASMMI	0.1 [−0.2–0.3]	0.0 [−0.1–4.3]	0.243
∆HG	1.7 [−1.2–4.3]	0.2 [−4.2–3.2]	0.764

Data are reported in median and interquartile range (25th, 75th percentiles). *p*-values refer to the Mann–Whitney U test. mBI, modified Barthel Index; FMA-UE, Fugl-Meyer assessment -Upper Extremity; MI-LE, Motricity index-lower extremity; MI-UE, Motricity index -upper extremity; MOCA, Montreal Cognitive Assessment; ASMMI, Appendicular Skeletal Muscle Mass Index; HG, hand grip strength. **p-value < 0.01.

## 4 Discussion

To our knowledge, this is the first study that explored the significant impact of sarcopenia, diagnosed by the EWGSOP2 criteria, on the functional and cognitive recovery in a cohort of Caucasian subacute post-stroke patients following a structured rehabilitation program. At admission, sarcopenic patients not only had a poorer nutritional status but also exhibited reduced independence in ADL, impaired upper limb performance, decreased strength in both upper and lower limbs, as well as diminished cognitive function and ambulation compared to non-sarcopenic patients.

Despite these baseline disadvantages, rehabilitation led to improvements across all functional and cognitive outcomes in both groups. However, sarcopenic patients after rehabilitation continued to show lower scores in independence in ADL and functional ambulation. Although gains in limb performance, strength, and cognitive abilities following rehabilitation were comparable between groups, a markedly lower recovery of independence in ADL was found in sarcopenic individuals compared to their non-sarcopenic counterparts. Notably, these differences persisted even after comparing sarcopenic and non-sarcopenic patients with similar baseline characteristics, confirming the detrimental effect of muscle wasting on the recovery of functional abilities after stroke.

In this study, sarcopenia was diagnosed in 28% of the enrolled patients, with almost all sarcopenic patients with severe sarcopenia. The prevalence of sarcopenia aligns with findings from previous studies on sarcopenia in subacute post-stroke patients, where rates range between 28% and 53% depending on the study and diagnostic criteria used (10, 13, 15, 25, 28, 50, 51). The high prevalence of severe sarcopenia in our cohort is likely attributable to the marked mobility impairments of the patients, many of whom had difficulty walking or were bedridden at admission—both factors that contribute to the deterioration of muscle mass and strength (52, 53).

Upon admission to the rehabilitation center, sarcopenic patients exhibited impaired nutritional status and compromised hematochemical parameters compared to their non-sarcopenic counterparts, confirming our preliminary study (28). Specifically, they had lower levels of nutritional indices, including serum albumin, GNRI, PhA, and MNA-SF®, further reinforcing the well-established association between sarcopenia and malnutrition (1, 24). These findings are in accordance with several previous studies on post-stroke sarcopenic patients, which similarly showed lower serum albumin (14, 54, 55), lower GNRI (28, 55), lower PhA (28, 36, 56–58) and lower MNA-SF® (11, 14). Additionally, we observed a higher prevalence of dysphagia among sarcopenic patients, consistent with prior research confirming the relationship between sarcopenia and dysphagia in post-stroke populations (10, 14). Sarcopenic patients also exhibited lower serum creatinine levels, suggesting its role as a potential biomarker for reduced muscle mass and sarcopenia (59, 60).

The significant baseline disparity observed between sarcopenic and non-sarcopenic individuals across all assessed functional and cognitive outcome measures, is a crucial point for rehabilitation care. Sarcopenic patients, in fact, had lower independence in ADL, reduced upper limb performance, decreased limbs strength, impaired cognitive abilities, and diminished functional ambulation with respect to non-sarcopenic individuals. The multidimensional assessments adopted in this study—integrating global functional assessments such as mBI and FAC with detailed evaluations of specific domains, including FMA-UE, MI-LE, and MI-UE, alongside cognitive impairment assessment using MoCA—allowed for an accurate characterization of the disadvantageous condition that sarcopenic patients experienced before to start the rehabilitation training. Studies in Asian populations examined such impact of sarcopenia on certain baseline functional outcomes in subacute post-stroke patients. A retrospective study (10) and one cross sectional study (11) showed lower ADL independence scores among sarcopenic patients at the onset of rehabilitation, while two other longitudinal studies with smaller sample did not identify baseline differences in ADL independence or functional ambulation between sarcopenic and non-sarcopenic groups (15, 16). The relationship between cognitive impairment and sarcopenia has been previously investigated reporting inconsistent results: while some research identified cognitive disparities between sarcopenic and non-sarcopenic individuals (10, 13), other studies did not confirm these differences (14, 26). However, it is important to note that these studies relied on the cognitive domain of the FIM, which may lack the sensitivity and comprehensiveness in detecting cognitive impairment assessed by the MoCA (61).

One of the key findings of this study is that, despite sarcopenic patients were more functionally and cognitively impaired than non-sarcopenic subjects at baseline, both groups improved in independence in ADLs, upper limb performance, limb strength, ambulation, and cognitive abilities by the end of rehabilitation. Notably, although the extent of recovery in upper limb performance, limb strength, and cognitive abilities—as assessed with ∆FMA-UE, ∆MI-LE, ∆MI-UE, and ∆MoCA—was comparable between the two groups, sarcopenic patients showed lower gains in ADL independence and functional ambulation. Specifically, they exhibited a more limited increase in mBI scores (∆mBI) at the end of rehabilitation, even when comparing sarcopenic and non-sarcopenic patients with similar baseline characteristics, confirming the results of our previous study (28). Additionally, a greater number of non-sarcopenic patients reached higher FAC levels compared to sarcopenic patients, who instead demonstrated reduced walking ability and increased reliance on assistance for ambulation. These results align with previous studies conducted on Asian post-stroke populations, which similarly reported associations between sarcopenia and reduced improvements in ADL independence and functional ambulation after rehabilitation (15, 16).

Our findings highlight the significant impact of sarcopenia on rehabilitation outcomes in post-stroke patients. The structured rehabilitation treatment allowed to all patients enrolled similar improvement of abilities in each district function domains, in line with the International Classification of Functioning, Disability and Health (ICF) (62). However, even if upper limb performance, limb strength, and cognitive abilities showed comparable improvements in both groups, sarcopenic patients reached lower scores, consistently with lower scores measured at admission. For this reason, after the rehabilitation, their daily living skills or walking capabilities remained considerably more limited.

Moreover, our findings indicate that the rehabilitation programs even when leading to partial functional improvements, may not adequately target the specific muscular deficits observed in sarcopenic individuals, which stem from reduced muscle mass and strength. Growing evidence from the literature strongly supports the implementation of multimodal, tailored interventions—encompassing dietary management, nutritional supplementation, and resistance training—in post-stroke individuals to mitigate sarcopenia and promote improvements in muscle mass and strength (63, 64). In particular, the combination of progressive resistance or task-oriented strength training with high-quality dietary patterns rich in fruits, vegetables, and unsaturated fats, together with adequate protein intake (1.2–1.5 g/kg/day, preferably from leucine-enriched sources) and optimal hydration, can effectively stimulate muscle protein synthesis, attenuate inflammation, and improve muscle strength and function (23, 63). These improvements may contribute to a better recovery after rehabilitation in sarcopenic individuals, as demonstrated from studies which showed that increases in muscle mass and strength are positively associated with higher functional recovery and independence levels at discharge in patients with stroke undergoing rehabilitation (65, 66).

The strengths of this study include its longitudinal design, the standardized rehabilitation intervention across all groups, and the comprehensive assessment framework. Notably, this study represents the first to investigate the impact of sarcopenia—defined according to the updated EWGSOP2 criteria—on both functional and cognitive recovery in a cohort of Caucasian post-stroke patients, thereby addressing a major gap in the existing literature, which to date has been almost entirely based on Asian cohorts. In fact, population-specific is crucial to improve the clinical applicability and global harmonization of sarcopenia research. Such a need arises because ethnic differences in body composition, dietary habits, and lifestyle patterns, which differ markedly between Asian and Caucasian populations, may substantially influence both the development and the functional consequences of sarcopenia (67). Compared with Asians, Caucasians typically exhibit higher BMI, lower body fat, greater absolute muscle mass, and distinct patterns of muscle loss with aging, which may modify both the prevalence and the clinical expression of sarcopenia (18, 68, 69). Consequently, the diagnostic criteria tools developed by the AWGS and the EWGSOP2 differ in their reference values, further complicating cross-ethnic comparisons and highlighting the need for region-specific evidence. In addition, ethnic-specific differences in dietary and nutritional patterns may also differentially affect the development of sarcopenia, as well as its impact on post-stroke recovery outcomes (22–24). Therefore, the inclusion of a Caucasian cohort of post-stroke patients, evaluated with EWGSOP2 criteria, not only fills a critical gap in the existing literature but also provides population-specific insights in sarcopenia research within the European context.

This study is limited by its single-center design and relatively small sample size, as the analysis of sarcopenia’s impact was secondary, and the study was not originally powered or designed for between-group comparisons. Further investigations in a large cohort of subacute post-stroke patients through a multicentre study (trial registered at ClinicalTrials.gov under the identifier NCT06547827) will allow us to analyze more precisely the association between sarcopenia and rehabilitation outcome, To this regard it will be possible to perform stratified analyses by muscle strength and muscle mass, to explore potential dose–response relationships between the degree of muscle impairment and recovery outcomes after stroke. Moreover, further research is needed to explore the impact of personalized nutritional strategies on muscle function and recovery in sarcopenic patients.

## 5 Conclusion

This study demonstrated for the first time that sarcopenia diagnosed according to EWGSOP2 guidelines significantly impacts the recovery of a Caucasian cohort of subacute post-stroke patients undergoing rehabilitation, addressing an important gap in the literature, focused mainly on Asian populations. Sarcopenic individuals began rehabilitation treatment with impaired functional abilities, limb performance, limb strength, and cognitive function. Although rehabilitation program led to significant improvements respect to baseline, sarcopenic patients reached lower results in these outcomes compared to non-sarcopenic counterparts. In particular, their recovery in ambulation and ADL independence were more limited compared, highlighting the persistent impact of muscle wasting on overall functional recovery. These findings underscore the importance of early identification and targeted interventions for sarcopenia in stroke rehabilitation programs to enhance recovery outcomes.

## Data Availability

The data supporting the findings of this study are available from the corresponding author upon reasonable request.
