# Supplementary material for: Impact of sarcopenia on functional and cognitive recovery in Caucasian post-stroke patients following rehabilitation
Source: Front Nutr. 2025 Dec 17;12:1694609. doi: 10.3389/fnut.2025.1694609 (PMC12753416; doi:10.3389/fnut.2025.1694609)
Supplement: Supplementary file 1 [file Data_Sheet_1.docx]

Supplementary Material

# Supplementary Figures


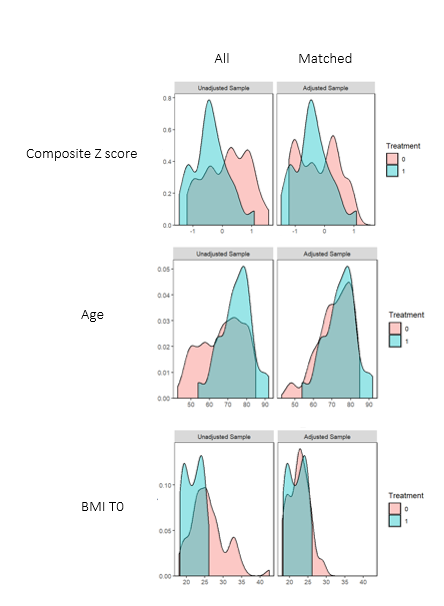


Supplementary Figure 1. Density plots of covariates before (left) and after (right) matching. Covariates fitted in the Propensity Score Matching analysis were: Composite Z-score (calculated as mean of the Z-scores of the following evaluation at T0: modified Barthel Index, Fugl-Meyer Assessment -Upper Extremity, Motricity Index-Lower Extremity, Motricity Index -Upper Extremity; Montreal Cognitive Assessment), Age and Body Mass Index at T0 (BMI at T0). Sky-blue area under curves represent the distributions for sarcopenic patients, pink area under curves represent the distributions for non-sarcopenic patients.

# Supplementary Tables

Supplementary Table 1. Baseline characteristics of sarcopenic (n=23) and non-sarcopenic (n=23) participants after Propensity Score Matching analysis. Data are reported in mean ± standard deviation for normally distributed data, median and interquartile range (25th, 75th percentiles) for non-normally distributed data, or number and percentage (%) for categorial variables. p-values refer to the Mann–Whitney U test or the chi-squared test, as appropriate. *p-value < 0.05.

| **Baseline Characteristics** | **Sarcopenic**  **n= 23** | **Non-sarcopenic**  **n= 23** | ***p-Value*** |
| --- | --- | --- | --- |
| Age | 75 [71–80] | 73 [67–69] | 0.391 |
| *Gender* |  |  |  |
| Women | 14 (61%) | 14 (61%) | 1.000 |
| Men | 9 (39 %) | 9 (39 %) |  |
| *Anthropometric measurements* |  |  |  |
| Weight (kg) | 56.8 [51.8–64.8] | 60.6 [54.8–66.4] | 0.503 |
| Height (m) | 1.62 [1.60–1.69] | 1.64 [1.55–1.71] | 0.878 |
| BMI (kg/m^2^) | 22.0 [19.7–24.1] | 22.7 [20.4–24.4] | 0.538 |
| *Index stroke type* |  |  |  |
| Ischemic | 21 (91%) | 15 (65%) | 0.032* |
| Hemorrhagic | 2 (9%) | 8 (35%) |  |
| Smoking habit (smokers and ex-smokers) | 10 (45%) | 9 (41%) | 0.761 |
| *Comorbidities* |  |  |  |
| Hypertension | 17 (74%) | 21 (91%) | 0.120 |
| Type 2 Diabetes | 4 (17%) | 9 (39%) | 0.102 |
| Dyslipidemia | 7 (30%) | 9 (39%) | 0.536 |
| Heart disease | 2 (9%) | 2 (9%) | 1.000 |
| Dysphagia | 14 (61%) | 9 (40%) | 0.140 |
| *Cumulative Illness Rating Scale (CIRS)* |  |  |  |
| CIRS severity | 2.2 [2.1–2.5] | 2.2 [2.0–2.4] | 0.371 |
| CIRS comorbidity | 5.0 [4.5–6.0] | 6.0 [5.0–7.0] | 0.275 |
| Days from stroke onset to enrollment | 91 [76–101] | 97 [77–135 | 0.373 |
| *Hematochemical Analyses* |  |  |  |
| Glucose (mg/dL) | 97 [85–130] | 99 [80–126] | 0.663 |
| Cholesterol (mg/dL) | 105 [87–140] | 132 [125–157] | 0.011* |
| HDL Cholesterol (mg/dL) | 49 [42–64] | 57 [49–69] | 0.248 |
| Triglycerides (mg/dL) | 96 [73–123] | 115 [95–146] | 0.093 |
| Albumin (g/L) | 3.6 ± 0.6 | 3.8 ± 0.4 | 0.151 |
| Creatinine (mg/dL) | 0.9 [0.8–1.1] | 0.9 [0.8–1.1] | 0.636 |
| *Nutritional status* |  |  |  |
| GNRI | 97 ± 12 | 100 ± 8 | 0.322 |
| MNA-SF® | 6.2 ± 2.8 | 7.3 ± 1.7 | 0.049* |
| PhA (degree) | 4.0 [3.5–4.6] | 4.4 [3.9–5.5] | 0.063 |
| *Independence in Activity of daily living (ADL)* |  |  |  |
| Modified Barthel Index T0 (0-100) | 34 [28–43] | 39 [35–50] | 0.441 |
| *Upper limb performance* |  |  |  |
| Fugl-Meyer T0 (0-66) | 18 [4–42] | 12 [6–40] | 0.652 |
| *Upper and lower limbs strength* |  |  |  |
| Motricity Index Upper Extremities (1-100) | 29 [6–56] | 40 [6–65] | 0.233 |
| Motricity Index Lower Extremities (1-100) | 43 [15–53] | 48 [25–74] | 0.329 |
| Functional Ambulation Category (0-5) | 0 [0–0] | 1 [0–2] | 0.947 |
| Montreal Cognitive Assessment (0-30) | 17 [14–21] | 17 [14–21] | 0.059 |
| *Data are reported in mean ± standard deviation. p-values refer to the Mann-Whitney test.* | | | |

| **Functional and cognitive**  **outcomes** | **Sarcopenic**  **n= 23** | **Non-sarcopenic**  **n= 23** | ***p-Value*** |
| --- | --- | --- | --- |
| mBI T1 | 40 [27–57] | 57 [47–72] | 0.044* |
| FMA-UE T1 | 23 [5–46] | 30 [10–51] | 0.510 |
| MI-LE T1 | 43 [22–70] | 65 [36-81] | 0.128 |
| MI-UE T1 | 40 [10–66] | 48 [20–77] | 0.439 |
| MoCA T1 | 20 [16–24] | 20 [17–23] | 0.921 |
| FAC T1 | 0 [0–1] | 3 [1–3] | 0.010* |
| *Data are reported in mean ± standard deviation. p-values refer to the Mann–Whitney U test.*  *mBI, Modified Barthel Index; FMA-UE, Fugl-Meyer Assessment -Upper Extremity; MI-LE, Motricity Index-Lower Extremity; MI-UE, Motricity Index -Upper Extremity; MoCA, Montreal Cognitive Assessment; FAC, Functional Ambulation Category.* | | | |

Supplementary Table 2. Functional and cognitive outcomes differences between sarcopenic (n=23) and non-sarcopenic (n=23) participants after Propensity Score Matching analysis. Data are reported in median and interquartile range (25th, 75th percentiles). P-values refer to the Mann–Whitney U test. *p-value < 0.05.
